# A consensus statement on child and family health during the COVID-19 pandemic and recommendations for post-pandemic recovery and re-build

**DOI:** 10.3389/frcha.2025.1520291

**Published:** 2025-01-29

**Authors:** Caroline A. B. Redhead, Sergio A. Silverio, Elana Payne, Mari Greenfield, Sara M. Barnett, Anna Chiumento, Beth Holder, Helen Skirrow, Ofelia Torres, Carmen Power, Staci M. Weiss, Laura A. Magee, Soo Downe, Lucy Frith, Claire Cameron

**Affiliations:** ^1^ Centre for Social Ethics and Policy, Department of Law, The University of Manchester, Manchester, United Kingdom; ^2^ Department of Women & Children’s Health, School of Life Course & Population Sciences, King’s College London, London, United Kingdom; ^3^ Department of Psychology, Institute of Population Health, University of Liverpool, Liverpool, United Kingdom; ^4^ Faculty of Wellbeing, Education & Language Studies, The Open University, Milton Keynes, United Kingdom; ^5^ Faculty of Medicine, Institute of Reproductive and Developmental Biology, Imperial College London, London, United Kingdom; ^6^ School of Social and Political Science, The University of Edinburgh, Edinburgh, United Kingdom; ^7^ School of Public Health, Faculty of Medicine, Imperial College London, London, United Kingdom; ^8^ Independent Researcher, Bristol, United Kingdom; ^9^ School of Psychology, University of Roehampton London, London, United Kingdom; ^10^ School of Nursing and Midwifery, University of Central Lancashire, Preston, United Kingdom; ^11^ Thomas Coram Research Unit, Social Research Institute, University College London, London, United Kingdom

**Keywords:** consensus statement, COVID-19, children's services, children and families, relationality

## Abstract

**Introduction:**

As health systems struggled to respond to the catastrophic effects of SARS-CoV-2, infection prevention and control measures significantly impacted on the delivery of non-COVID children's and family health services. The prioritisation of public health measures significantly impacted supportive relationships, revealed their importance for both mental and physical health and well-being. Drawing on findings from an expansive national collaboration, and with the well-being of children and young people in mind, we make recommendations here for post-pandemic recovery and re-build.

**Methods:**

This consensus statement is derived from a cross-disciplinary collaboration of experts. Working together discursively, we have synthesised evidence from collaborative research in child and family health during the COVID-19 pandemic. We have identified and agreed priorities areas for both action and learning, which we present as recommendations for research, healthcare practice, and policy.

**Results:**

The synthesis led to immediate recommendations grouped around what to retain and what to remove from “pandemic” provision and what to reinstate from pre-pandemic, healthcare provision in these services. Longer-term recommendations for action were also made. Those relevant to children's well-being concern equity and relational healthcare.

**Discussion:**

The documented evidence-base of the effects of the pandemic on children's and family services is growing, providing foundations for the post-pandemic recovery and re-setting of child and family health services and care provision. Recommendations contribute to services better aligning with the values of equity and relational healthcare, whilst providing wider consideration of care and support for children and families in usual vs. extra-ordinary health system shock circumstances.

## 1 Introduction

The COVID-19 pandemic represented an unprecedented global health system shock. Between January 2020 and May 2023, when the WHO concluded that COVID-19 no longer constituted a public health emergency of international concern (1), concerns about mortality and spread of the novel coronavirus prompted a global, co-ordinated implementation of social and physical distancing restrictions. Meanwhile, research efforts turned towards vaccine development (2), understanding the health system shock, its implications for healthcare decision-making (3) and the possible ramifications for short-, medium-, and long-term health, especially as the world braced for the impact of the inevitable rise in mental health issues caused or exacerbated by the virus, and associated fears, bereavements, and restrictions (4). Social and physical distancing restrictions interrupted child and family health services and routine perinatal care (5, 6) and strict requirements relating to the wearing of personal protective equipment (PPE) in hospitals literally changed the face of healthcare from a child's perspective. Worryingly, social and physical distancing restrictions also led to families being isolated at home (7), increased instances of child neglect, child abuse, and domestic abuse (8), and a deterioration in metal health amongst children and adolescents (9).

The full extent of the longer-term, intergenerational impacts of the pandemic on children and families is yet fully to be realised and may take years to be understood completely. It is clear, however, that from a systemic perspective, the health of the population engaging with child and family health services has, and continues to be, deleteriously impacted by the pandemic (10). These abrupt changes in lifestyle, service provision, family support networks, and nursery closures were associated with an increase in parental stress and mental health, leading to poorer parent-child bonding, with potentially long-term impacts on children's wellbeing and development (11).

In this article, we present a consensus statement developed from research undertaken during the COVID-19 pandemic to investigate its impact on child and family healthcare services, with a broad interpretation of “health”. Our research has engaged with families from preconception to the pregnancy-to-pre-school lifestages, as well as with healthcare professionals working in associated healthcare services. The empirical evidence collected has been synthesised to inform the consensus and to underpin our recommendations. We suggest that some changes made to child and family health services during the pandemic should be retained and others should be removed from care provision as we move through the period of post-pandemic recovery and re-build. We also identify some services which were offered routinely pre-pandemic and then were withdrawn, but that should, as soon as possible, be reinstated. Finally, with the well-being of children and families in mind, we offer suggestions on longer-term recommendations for practice in both “usual” and “extra-ordinary” circumstances.

## 2 Methods

**Figure 1:**
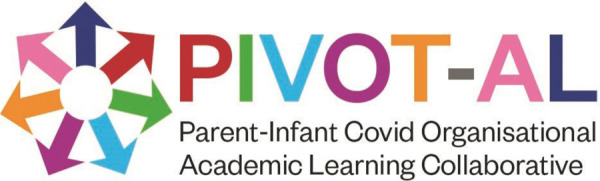
The PIVOT-AL logo.

A consensus statement on perinatal mental health has already been published by some members of this group (12), within which we have published details of our network and a detailed methodology [see also (13)]. In brief, a group of more than 60 researchers, academics, policy makers, and members of third sector organisations from more than 25 institutions formed a national collaborative called The Parent-Infant Covid Organisational Academic Learning Collaborative (PIVOT-AL; Figure 1). Our research was iteratively synthesised during the pandemic, and our regular on-line meetings informed a dissemination event held at The Royal Society of Medicine (RSM) in London on 22 September 2022 (funded by the Society for Reproductive and Infant Psychology, via a Research Development Workshop Grant (ref:- SRIP/DWA/01). At The RSM event, a formal synthesis of a spectrum of evidence relating to maternity and child healthcare services was presented and discussed. This consensus statement summarises of these evidence-based deliberations, identifying priorities for future research, policy, and healthcare practice.

## 3 Available evidence

Various teams from within the PIVOT-AL Collaborative have focused efforts on attempting to understand the impact of the pandemic health system shock and associated reconfigurations of service provision on child and family health services, widely construed. We have amassed a cross-disciplinary evidence base, drawn from research investigations including the impact of the pandemic on maternity services, neonatology and hospital-based paediatric services as well as the place-based societal impacts on families and young children. Much of the research takes a family-centred approach, exploring, for instance, the impact of vaccination, of changed hospital service provision for newborns with significant complications and of long-stay paediatric patients (12), and diminished support for important issues that new parents may need help with such as parent-infant bonding (15, 16), as well as more generally for the generation of pandemic-born babies who lived their first months and their early years during, or in the wake of, the pandemic (17, 18). Our research teams also studied the ethical challenges resulting from “re-setting” non-pandemic maternity and children's services to run concurrently with pandemic services. The impacts of these ethical dilemmas affected children, families and healthcare professionals in different ways (19–21). Critically, they all relate to the importance of parent-infant relationships as well as those with child and family health services, and this common factor underpins our recommendations for the future of these services, as set out below.

### 3.1 Re-organising child and family healthcare services

In re-organising non-COVID-19 healthcare services alongside a continuing pandemic response, ethical considerations had to underpin healthcare decision-makers' choices about integrating infection prevention and control measures into routine healthcare practice. New kinds of ethical issues and dilemmas arose as assessments were made as to how best to balance patients' and families' access to healthcare services with the protection of both hospital communities and the wider public from COVID-19. The ethical challenges of (re)organising healthcare services to facilitate the continued provision of maternity and paediatric services during COVID-19 was the focus of the multi-disciplinary Reset Ethics Project, which found distinct and different ethical issues are associated with acute and “reset” phases of a pandemic (5).

Qualitative data, collected as part of the Reset Project between November 2020 and July 2021, indicated significant challenges were encountered by healthcare professionals in their struggle to comply with (sometimes rapidly changing) infection prevention and control measures and, at the same time, offer the level of patient care they felt their personal standards and professional obligations required. In decision-making about re-organising maternity and paediatric services, engagement with ethical principles was found to be *ethics-lite*, with sources mentioning principles in passing rather than explicitly applying them (3). The mandating of personal protective equipment (PPE), the social distancing requirements and the measures imposed to reduce footfall within hospitals (such as banning birth partners and allowing only one parent at a time to be with a hospitalised child) were experienced by healthcare professionals as barriers to their engagement with patients and their families; barriers which impeded on the creation and development of supportive, caring relationships with family, friends, and healthcare professionals (19). The Reset data indicate that, for healthcare professionals, offering care as part of a relational interaction was experienced as an ethically important dimension of healthcare delivery (20, 21).

Further qualitative data from the Changing Children's Healthcare Study in London demonstrated racial and ethnic discrimination amongst children's healthcare staff, with micro-aggressions occuring between ethnic in-groups and out-groups who were otherwise meant to be working together in the most difficult of times (22). The research also found responsibility for health and psychological well-being was being discharged to individual staff rather than clinical management (22). Neither maternity care staff (23), children's healthcare staff were not prepared for the gravity of the pandemic health system shock. However, the latter were more likely to break the instituted rules to provide care for the children in their clinics and their wider family units, than their colleagues in maternity care (24, 25).

### 3.2 Child and family health and social care systems and networks

PIVOT-AL researchers investigating place-based impacts of the pandemic worked with families and young children in Tower Hamlets and Newham (boroughs of London situated on the north bank of the River Thames and of the City of London), and Bradford (a city in the North of England), both places with substantial south Asian populations (among other ethnic groups) and high levels of poverty. Low-income families were least likely to be employed, own their own home, or have sufficient indoor space or access to outdoor space. South Asian parents and fathers of all ethnicities were found to have more significant levels of depression, which was also exacerbated by a lack of access to outside space (26). Pre-pandemic uneven distribution of material assets was exacerbated for some ethnic groups and housing quality was poor. For those unemployed and on furlough, in terms of time available for children and family life, the experience was quite different from that of those working from (or away from) home. The research across the three areas found that the pandemic exacerbated existing inequalities, having a greater impact on those already vulnerable. Financial insecurity, loneliness, levels of social support, and location of residency were all associated with clinically important depression and anxiety during the pandemic (27). Work led by the Parent-Infant Foundation echoed these findings, suggesting that the pandemic exacerbated experiences of isolation, a lack of support and mental health challenges. Mothers were also found to have struggled to initiate breastfeeding during lockdown restrictions without in-person support. Whilst caregiving interactions were found not to have suffered from a reduction in social exposure, some babies born in lockdowns did demonstrate lower responsiveness to sensory stimuli, although mediated through caregiving quality and other social interactions (17, 18). We do not yet know how parent perceptions of their infants as “COVID babies” might have consequences on parenting, parent-child attachment, parent and child mental health and school adjustment. Longitudinal research in the coming years will reveal if the significance of the lack of social exposure and increased stress has an enduring impact on parents and children; whilst the severe reduction in vital services may have already led to long-term negative impacts on this generation of babies and young children (28).

The disruption of antenatal care and routine vaccination schedules for mothers and infants during the pandemic resulted in lower vaccination rates (29). PIVOT-AL researchers found that these disruptions generated confusion and access issues, with a high proportion of changed appointments, and reported fears about attending healthcare settings for routine vaccinations (30). Women from ethnic-minorities and lower-income households were less likely to be vaccinated, and minority-ethnic women were more likely to report access problems and feeling less safe attending vaccinations for both themselves and their babies (30). When it came to roll-out of COVID-19 vaccination, willingness to be vaccinated during pregnancy was significantly lower, and this reluctance was significantly higher in minority-ethnic women and those of low-income (31). Concerns around the safety of the vaccine for pregnant or breastfeeding women, and for their babies was common, sometimes alongside wider feelings of mistrust around vaccines. A lack of data, the speed of vaccine development and worry about side effects were the three main themes concerning perceived safety of COVID-10 vaccination in pregnancy (31). Pregnant and postnatal women were reported to have seen the COVID-19 vaccination efforts as rushed, and to have expressed strong concerns around the safety of the vaccine for pregnant or breastfeeding women, and for their babies (32). Vaccine misinformation was found to have spread quickly, and was thought to be problematic for the encouragement of both routine and COVID-19 vaccination uptake (33).

## 4 Discussion of recommendations

Our recommendations below draw on our synthesis of the research carried out by teams working across the PIVOT-AL Collaborative and identify pandemic-related changes to service provision which represented risks to child and family health. Our recommendations are broadly divided into three key areas of focus. Thinking of changes made to service delivery during the pandemic, we make recommendations as to (i) those adaptations which should be retained as innovative ways for delivering care; (ii) those elements which were stopped and should be reinstated to ensure safety, accessibility, and/or satisfaction with care; and (iii) those aspects of reconfigured care which should be removed from post-pandemic service provision as they have been not been found to add value, or to result in negative experiences of care. We also make longer-term recommendations for focused efforts amongst health service policy makers and practitioners could focus their efforts to better protect the well-being of parents, children, and families. These are not set out in terms of rank or relative importance.

### 4.1 What to retain

The adoption of technological solutions to facilitate remote access to healthcare services was a notable feature of pandemic healthcare provision. However, remote care must be offered in-line with clinical decision-making around safety and appropriateness for children and families. Maintenance of (at least some) virtual or remote care provision, or at least the option to attend some appoints virtually, was generally seen by both service users and healthcare professionals to be acceptable and, in some cases, preferable. A must, however, is the inclusion of birth partners and family members (to whose presence service users—including children, where appropriate—consent) in all child and family healthcare services. The pandemic—on occasion—allowed for creativity, adaptability, and flexibility to innovate from the bottom-up, sometimes rapidly. This agility, coupled, where possible, with patient and public involvement and engagement, should, where necessary, continue to be facilitated in post-pandemic service provision.

### 4.2 What to reinstate

While we have recommended continuing to facilitate some of the agility which characterised pandemic innovation, we note that, in many cases, consultation and engagement was lacking. At a system level, reinstating time for processing and ethical reflection on new directives for service delivery is important. Healthcare professionals and service users (including children, where appropriate) should be involved in discussion and decision-making across all aspects of child and family healthcare (3, 24, 25).

Pandemic infection prevention and control measures often displaced (or, at best, reduced) healthcare professionals' clinical autonomy (19). Where the resulting care fell short of what healthcare professionals considered their professional ethical duties obliged them to provide, some experienced moral distress (19). We, thus, recommend that, even in the context of a pandemic, healthcare professionals’ autonomy and professional judgment as to what constitutes safe care is respected. This would include permitting infection prevention and control measures to be flexed where other considerations are at stake such as, for example, offering in-person care in preference to virtual care where there is a concern about domestic or child abuse (34). Our research suggests that this will allay staff anxieties, both about falling short of offering the care they feel (professionally or personally) morally obliged to deliver and about “breaking” infection prevention rules by offering what would usually be healthcare and family support.

Finally, an important recommendation is the permanent re-introduction family members to neonatal and children's wards. In many specialist children's services, family members are considered a valuable part of the team and preventing or limiting their access during the pandemic caused significant harm both to families and to the healthcare professionals providing care (19–21). It is clear from our evidence base that relational care is morally significant to healthcare professionals and important for patients and families (20, 21). This is true across child and family health services, and re-establishing high quality, joined-up provision of compassionate care to children and families is crucial to their ongoing health and well-being. All of this, however, requires the availability of face-to-face provision of care and support—in hospitals and healthcare settings, and in the community—the removal of which has been repeatedly criticised. Ultimately, face-to-face, compassionate care should be offered in all cases and sometimes, for example where domestic violence is suspected, be mandatory across family services. It is clear that a hospitalised child's parents were considered an important part of healthcare team prior to the pandemic (20). Thus, our recommendation is not simply that family members should be welcomed back into child and family healthcare services but that the importance of their role should explicitly be recognised. Excluding them to reduce an infection risk opens the door to the possibility of equally significant emotional and psychological risks, which might have intergenerational consequences.

### 4.3 What to remove

Firstly, blanket or “one size fits all” policies should not be rolled-out across health and community services without consideration of variation in demographic need or accessibility to essential support services. This was clearly a rapid-response approach used in acute stages when the virus was not well understood, but, as time passed, the continued displacement of moral, compassionate and relational care was damaging to children, families and healthcare professionals (19, 20, 35). A narrow understanding of risk focused on the prevention of infection ignored risks linked to mental health and psychological safety, opening the door to different but equally significant consequences. These include severe mental health episodes, domestic abuse and violence, and suicide; all of which may impact parents' ability to bond with their child, with serious consequences for infant outcomes (11).

We also note the crucial importance to families of effective, trustworthy communication across maternity and child development infrastructure. Confusing and conflicting messaging between Government organisations, the Royal Colleges, individual Trusts, and other Learned Academies, was (and, to some extent continues to be) an issue. When national public health messaging is necessary, disinformation and/or conflicting information must be stopped as a matter of utmost importance (3, 36, 37). Messaging must be consistent from policy to practice and must include operationalisable ethical frameworks and guidance for healthcare professionals (3). Policy-makers and healthcare professionals must be agile enough to interpret and implement change in a uniform way.

## 5 Longer-term recommendations and future directions

### 5.1 Equity, ethics, and relational healthcare

Equitable, relational, compassionate care should be offered to all, with special consideration made for populations who struggle to access healthcare (19, 21, 38). This includes those living with high levels of social complexity or in areas with high levels of social deprivation (39), who may mistrust the NHS and wider social care systems (23) or are generally underserved by health and care systems (40). It would also be prudent to attend to the established relationship between parental, child health, and wider family health. This should acknowledge the reciprocal nature of the caregiver-infant mental health outcomes (41) and ensure healthcare professionals are working holistically (35) and with wider social and community services to enable a proactive model of support which facilitates intervention before families reach crisis point (28).

Protecting healthcare professionals' emotional well-being and capacity (20, 21), protecting against redeployment in times of health system stress or shock, and arguing for greater representation of minoritized staff, is recommended across all maternity and children's healthcare services (22). Better integration of physical and mental health care is also required (42). Access to common outdoor spaces and anticipated social spaces (e.g., infant care groups) are acutely important for supporting mental health during the parenting journey; their access should be given special consideration in light of protecting the physical health of the public.

### 5.2 A lifecourse approach to child and family health

Whilst this consensus statement focuses on research carried out during the pandemic in the United Kingdom, we would strongly recommend making comparisons with research from across the globe. An important next step would be for formal inter-cultural comparisons to be made. It is expected that recommendations could be derived from these comparisons and could therefore be synthesised. To ensure the future safety and security of children and their families over their lifecourse as they move on from the pandemic, we must also take into account the different resource available for child and family healthcare, especially in those countries where healthcare is not free-at-point-of-access (43); see also (44). These recommendations would then help the next generation of healthcare if acted upon, and could offer some plasticity to the healthcare systems caring for children and families in future health system shock situations (45).

Furthermore, it is imperative that the longer-term detrimental effects of specific aspects of countries' governments' pandemic-related responses are explored. For example, it is widely recognised that prolonged quarantine measures are detrimental, psychologically (7), however, “lockdowns” to prevent the spread of infection had many other effects, the impact of which are still being calculated and understood. For example, the food insecurity which came about as a result of restricted movement, decreased access to healthcare, and heightened, chronic, stressful situations (such as those spent unable to leave home, or in high-intensity hospital care settings), have already been highlighted as having the potential to have a long term impact on the lifecourse of a “child of the pandemic” (46). Moreover, parent-infant relationships were challenged during the pandemic, with parents—often mothers—having to juggle the needs of their work, their family units, and their children as well as their education (47). It has already been found that these pandemic-related stressors have led to poorer parental mental health overall (48) and we know poor parental mental health can have a detrimental effect on their children's lifecourse (49). Future research must also attempt to understand the effect of stressors such as the pandemic on the very worst outcomes ruptures between families and the children of those families, such as ambivalence, abandonment, abuse, and infanticide (50), the lifecourse health effects of which are devastating, long-lasting, and often incalculable.

## 6 Conclusion

We have set out in this statement a brief review of the empirical evidence amassed during the pandemic by research teams who came together under the PIVOT-AL Collaborative banner. While COVID-19 is now known to pose less of a risk to children and young people, it is arguable, as we have shown above, that both the immediate and longer-term impacts of pandemic decision-making in health services had, and continue to have, significant consequences for children and families. The pandemic has made dramatic changes to the fabric of health and care services. In order to promote the health and well-being children and families both in times of relative stability, and during times of global crisis, the health and child development services of the future must be resilient, adaptable, tensile, and plastic enough to weather the inevitable systemic shocks. Health services must also attend to the (ethical) significance of relationships. We have suggested that new ways of thinking are required to optimise the provision of these services going forward, and to prepare them for future shocks. These include those that can be anticipated (for instance, related to climate change) and those that are currently unforeseen. The evidence-base on the extent of the very worst outcomes possible for children and family health have yet to be totalled and may forever be incalculable, however, future research should address issues of intra-familial estrangement, harm, and rupture more directly to further improve child and family healthcare delivery in future health system shocks. To avoid current and future disaster, healthcare and child development decision- and policy-makers must push the boundaries of what is practically possible now and in the very near future in designing evidence based, equitable, relational and future-proofed child and family health services. We hope this statement will assist with such endeavours.
